# Emission Ratios for Ammonia and Formic Acid and Observations of Peroxy Acetyl Nitrate (PAN) and Ethylene in Biomass Burning Smoke as Seen by the Tropospheric Emission Spectrometer (TES)

**DOI:** 10.3390/atmos2040633

**Published:** 2011-11-09

**Authors:** Matthew J. Alvarado, Karen E. Cady-Pereira, Yaping Xiao, Dylan B. Millet, Vivienne H. Payne

**Affiliations:** 1 Atmospheric and Environmental Research (AER), Lexington, MA 02421, USA; 2 Department of Soil, Water, and Climate, University of Minnesota, St. Paul, MN 55455-0213, USA

**Keywords:** biomass burning, satellite, TES, formic acid, ammonia, PAN, ethylene

## Abstract

We use the Tropospheric Emission Spectrometer (TES) aboard the NASA Aura satellite to determine the concentrations of the trace gases ammonia (NH_3_) and formic acid (HCOOH) within boreal biomass burning plumes, and present the first detection of peroxy acetyl nitrate (PAN) and ethylene (C_2_H_4_) by TES. We focus on two fresh Canadian plumes observed by TES in the summer of 2008 as part of the Arctic Research of the Composition of the Troposphere from Aircraft and Satellites (ARCTAS-B) campaign. We use TES retrievals of NH_3_ and HCOOH within the smoke plumes to calculate their emission ratios (1.0% ± 0.5% and 0.31% ± 0.21%, respectively) relative to CO for these Canadian fires. The TES derived emission ratios for these gases agree well with previous aircraft and satellite estimates, and can complement ground-based studies that have greater surface sensitivity. We find that TES observes PAN mixing ratios of ~2 ppb within these mid-tropospheric boreal biomass burning plumes when the average cloud optical depth is low (<0.1) and that TES can detect C_2_H_4_ mixing ratios of ~2 ppb in fresh biomass burning smoke plumes.

## 1. Introduction

Biomass burning is the second largest source of trace gases to the global atmosphere and is an important part of the interannual variability of atmospheric composition [1–3]. Trace gases from biomass burning can contribute to the secondary chemical formation of aerosol particles and global tropospheric ozone, both of which impact upon climate and human health. However, the emissions of trace gases from biomass burning are highly uncertain. Emission factors for biomass burning are primarily based on airborne and ground field measurements and measurements from small fires conducted in laboratories. Laboratory studies have shown that emissions of trace gases and particles from biomass burning can vary widely based on the type of fuel burned as well as the phase of combustion (*i.e.*, whether the combustion is in the early “flaming” stages or the later “smoldering” stages) [4]. However, the size, fuel moisture, and combustion characteristics of laboratory fires may not be representative of large-scale wildfires. Aircraft and ground studies of biomass burning emissions can only sample a small number of fires infrequently, making it difficult to understand the impact that the regional variability of fuel type and combustion phase can have on biomass burning emissions. Satellite observations, with their extensive spatial and temporal coverage, provide the opportunity to sample a large number of fires in several different ecosystems, which will help to characterize the spatial and temporal variability of emissions within a region for use in models of atmospheric chemistry, air quality, and climate.

Recent investigations have focused on using nadir-viewing satellite observations to estimate biomass burning emissions of trace gases and particles and study their subsequent chemistry. Examples of these studies include: estimating the emission rate of fine particles with fire radiative power (FRP) and aerosol optical depth retrievals from the Moderate-Resolution Imaging Spectroradiometer (MODIS) [5,6]; estimating emissions of NO_x_ with MODIS FRP and tropospheric NO_2_ columns from the Ozone Monitoring Instrument (OMI) [7]; constraining emissions of CO using retrievals from the Measurements Of Pollution In The Troposphere (MOPITT) instrument, the Atmospheric Infrared Sounder (AIRS), the Scanning Imaging Absorption Spectrometer for Atmospheric Cartography (SCIAMACHY), and the Tropospheric Emission Spectrometer (TES) [8]; detecting several trace gases within smoke plumes using the Infrared Atmospheric Sounding Interferometer (IASI) [9,10]; and estimating the correlation between CO and O_3_ in smoke plumes with TES [11–13].

TES made multiple special observations during the summer of 2008 over eastern Siberia, the North Pacific, and North America as part of the Arctic Research of the Composition of the Troposphere from Aircraft and Satellites (ARCTAS-B) campaign [14]. This data set includes several observations of smoke plumes from boreal fires in Siberia and Canada. Alvarado *et al.* previously analyzed the correlation between TES retrievals of CO and O_3_ within these smoke plumes [12]. Here we use nadir observations from TES to determine the concentrations of the trace gases ammonia (NH_3_) and formic acid (HCOOH) within two boreal biomass burning plumes over Canada and determine the emission ratio of these gases relative to CO. The use of emission ratios relative to CO allows us to build on previous studies of emissions of CO from biomass burning [8] to estimate emissions of these less well-studied trace gases. We also present the first TES detections of peroxy acetyl nitrate (PAN), an important reservoir species of nitrogen oxides (NO_x_) that is formed chemically within biomass burning smoke plumes, and the first TES detection of ethylene (C_2_H_4_), a reactive hydrocarbon emitted by biomass burning.

Biomass burning is a significant source of NH_3_ [15]; other anthropogenic sources are livestock and chemical fertilizers, while natural sources include oceans, wild animal respiration, and soil microbial processes [16]. NH_3_ is an integral component of the nitrogen cycle. It can combine with acidic gases like H_2_SO_4_ and HNO_3_ to form secondary aerosol, which then can impact climate and human health. This reactivity leads to a very short lifetime (less than two weeks) and large temporal and spatial variability. Background summer ammonia mixing ratios in the United States can range from 0.05 to 47 ppbv [17]. *In situ* observations of atmospheric ammonia are sparse and infrequent, making satellite observations of tropospheric NH_3_ highly desirable. Worden *et al.* derived molar ratios of NH_3_ to CO in smoke plumes from forest fires near San Luis Obispo, California, on 15 August 1994 using the Airborne Emission Spectrometer (AES), the airborne prototype for TES [18]. Beer *et al.* reported the first satellite observations of boundary layer NH_3_ using the TES instrument aboard Aura [19]. Shephard *et al.* extended this work to a detailed strategy for retrieving NH_3_ using TES [20]. Pinder *et al.* showed that the TES NH_3_ retrievals were able to capture the spatial and seasonal variability of NH_3_ over eastern North Carolina and that the retrievals compared well with *in situ* surface observations of NH_3_ [21]. In addition, Clarisse *et al.* have used the nadir viewing IASI instrument to retrieve mixing ratios and global distributions of tropospheric NH_3_ [10,22].

Formic acid (HCOOH) is a significant contributor to the acidity of precipitation and is an important oxygenated volatile organic compound [23–25]. Formic acid is ubiquitous in the troposphere, with typical surface concentrations ranging from 0.1 ppbv for “clean” environments to over 10 ppbv in urban polluted environments (e.g., Table 1 of [26]). The HCOOH lifetime ranges from several hours in the boundary layer to a few weeks in the free troposphere with wet (precipitation) and dry deposition the primary sinks, and reaction with OH of lesser importance [27]. There is considerable uncertainty concerning the origin of formic acid in the atmosphere. Some identified HCOOH sources include biogenic emissions from vegetation and soils, emissions from motor vehicles, and secondary production from organic precursors [26–28].

Biomass burning is another major primary source of formic acid, with several airborne studies showing that secondary production of formic acid also takes place within the aging smoke plume as the initial organic gases in the smoke are oxidized [29]. Enhanced mixing ratios of formic acid were measured in TES prototype airborne measurements of western wildfires [18]. The limb-viewing Atmospheric Chemistry Experiment Fourier Transform Spectrometer (ACE-FTS) observed formic acid in young and aged biomass burning plumes in the upper troposphere and derived emission ratios for formic acid to CO [30,31]. Similarly, Grutter *et al.* used the limb-viewing Michelson Interferometer for Passive Atmospheric Sounding (MIPAS) to retrieve global distributions of formic acid in the upper troposphere and stratosphere [32]. Razavi *et al.* presented global distributions of formic acid retrieved using the nadir-viewing IASI instrument and showed that the retrieved formic acid is correlated with CO during the burning season in Brazil, the Congo, and Southeast Asia [33].

PAN is a thermally unstable reservoir for NO_x_ that can be transported over large distances before converting back into NO_x_, thereby altering ozone formation far downwind from the original source [34–36]. The primary NO_x_ emissions from biomass burning are rapidly converted to PAN within biomass burning plumes [12,37]. Satellite retrievals of PAN could provide substantial information on the fate of NO_x_ emitted by biomass burning in the atmosphere and the impact of these NO_x_ emissions on global tropospheric ozone. The limb-viewing sounders MIPAS [38] and ACE-FTS [39] and the nadir-viewing IASI instrument [9,10] have all previously identified PAN in biomass burning smoke, but this species had not been previously detected in TES spectra.

Ethylene (C_2_H_4_) is a reactive hydrocarbon that is emitted directly by biomass burning [3]. It has a short lifetime in the summer Arctic troposphere (14–35 h, [12]) due to rapid reaction with OH. As this rapid oxidation of ethylene impacts the ozone formation rate within young smoke plumes [40], better estimates of the emissions of ethylene from biomass burning could help to reduce the uncertainty in the impact of biomass burning on tropospheric ozone. Enhanced mixing ratios of ethylene were measured in TES prototype airborne measurements of western wildfires [18], and C_2_H_4_ has also been previously identified by ACE-FTS [39] and IASI [9,10], but it had not been previously detected in TES spectra.

Section 2 describes the methods we used to identify biomass burning plumes from TES spectra, to retrieve NH_3_ and HCOOH within the smoke plumes, and to detect PAN and C_2_H_4_ in TES spectra. Section 3 presents the results of this study and Section 4 summarizes our conclusions.

## 2. Methods

### 2.1. TES Special Observations During ARCTAS-B

TES is a nadir-viewing Fourier-transform infrared (FTIR) spectrometer aboard the NASA Aura spacecraft with a high spectral resolution of 0.06 cm^−1^ and a nadir footprint of 5.3 km × 8.3 km. Here we use Level 1B spectra (V003) for the 1B2 (950–1150 cm^−1^) and 2A1 (1100–1325 cm^−1^) bands of the TES instrument [41].

TES retrievals of trace gas profiles are based on an optimal estimation approach (with *a priori* constraints) that minimizes the differences between the TES Level 1B spectra and a radiative transfer calculation that uses absorption coefficients calculated with the line-by-line radiative transfer model LBLRTM [20,42–45]. Current Level 2 products from TES (V004) include retrieved profiles of CO, O_3_, H_2_O, HDO, and CH_4_. The NH_3_ retrieval discussed below will be included in the upcoming V005 of TES products, while the HCOOH retrieval discussed below is a prototype retrieval being developed at AER. The averaging kernel matrix of the retrieval gives the vertical sensitivity of the retrieved profile to the true profile, while the trace of the averaging kernel gives the degrees of freedom for signal (DOFS), which represents the number of independent pieces of information contained in the retrieval [44]. Cloud properties are retrieved by assuming single layer clouds with an effective optical depth that accounts for both cloud absorption and scattering [46]. Due to their small size (count median diameters of ~0.13 μm [47]), biomass burning aerosols are unlikely to significantly impact radiances in the thermal infrared regions detected by TES, and any impact from larger particles is accounted for by the retrieved effective cloud optical depth.

The TES special observations during ARCTAS-B included nadir observations over eastern Siberia, the North Pacific, and North America for every 0.4° latitude. Biomass burning plumes were identified following the procedure in Alvarado *et al.* [12], which we briefly outline here. We used maps of Level 3 daily AIRS retrievals of CO at 1° × 1° resolution to identify the transport of CO from major regions of boreal biomass burning. We then used TES Level 2 retrievals of CO (V003) to identify the corresponding plumes that were observed by TES. The CO retrievals for TES special observations between 15 June and 15 July 2008 were filtered for data quality as recommended in the TES Level 2 Data Users Guide [48]. In general, the retrievals had 1 DOFS below 250 hPa with the region of maximum sensitivity in the troposphere near 500 hPa. We defined a plume in the TES special observations as an area where the retrieved CO mixing ratio at 510 hPa exceeded 150 ppb. This threshold ensured that the CO retrievals were significantly different from the *a priori* values (~110 ppb). While this procedure will detect thick plumes that are transported between continents [49], it does not detect plumes near the surface (where the sensitivity is low) or very thin or dilute plumes. We then used HYSPLIT back-trajectories [50] to determine if the observed air masses came from boreal biomass burning regions in Siberia (17 plumes) and Canada (5 plumes). The CO and O_3_ retrievals for these plumes were analyzed by Alvarado *et al.* [12]. In this paper, we restrict our analysis to the fresh plumes from Canadian biomass burning.

### 2.2. NH_3_ Retrieval

**Figure 1.**
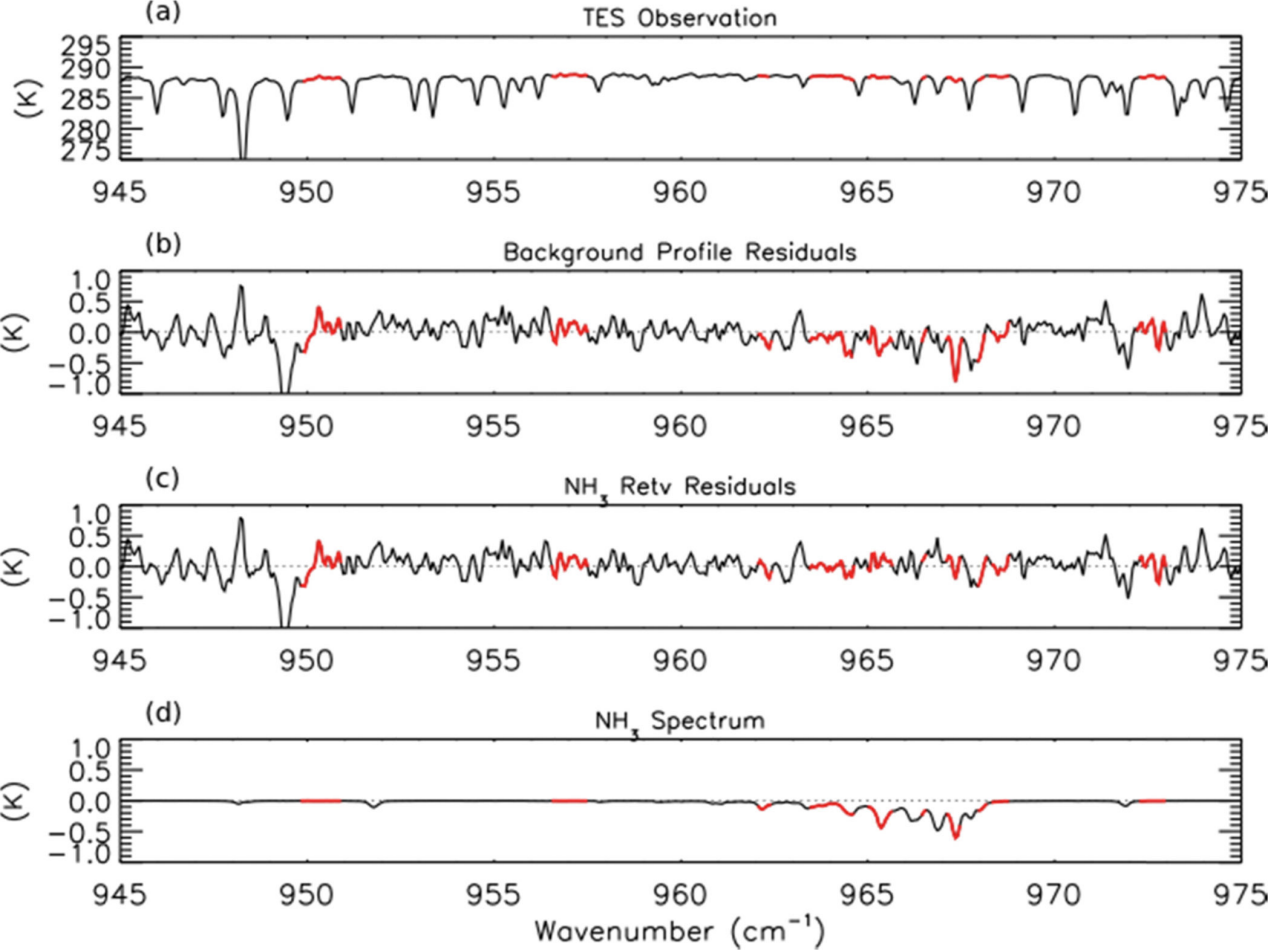
(**a**) Tropospheric Emission Spectrometer (TES) observed brightness temperature spectrum, (**b**) the background profile residuals, calculated as observed minus modeled spectrum for an unpolluted profile of NH_3_, (**c**) the residuals after retrieval of NH_3_, and (**d**) the modeled spectrum of NH_3_ for the TES scan at 53.32° N and 90.84° W on 1 July 2008 near a Canadian fire. The microwindows used in the NH_3_ retrieval are shown in red. Note that surface emissivity and temperature are adjusted during the retrieval, which requires microwindows outside of the NH_3_ absorption band (~960–970 cm^−1^).

NH_3_ retrievals were performed using TES Level 1B spectra (V003) [41] following the method of Shephard *et al.* [20], which has been implemented in V005 of the TES Level 2 products. The *a priori* profiles and covariance matrices for TES NH_3_ retrievals are derived from GEOS-Chem model simulations of the 2005 global distribution of NH_3_. Figure 1a shows an observed TES brightness temperature spectrum in the region of strong NH_3_ absorption for a scan of a fresh Canadian smoke plume. Figures 1b,c show the brightness temperature residuals (observed spectrum minus modeled spectrum) for the unpolluted background NH_3_ profile and the retrieved NH_3_ profile, respectively, while Figure 1d shows the modeled spectrum of NH_3_, calculated as the difference between the modeled spectrum including the retrieved NH_3_ profile and the modeled spectrum without any NH_3_. We can see a strong residual (−0.8 K) in the background profile spectrum that is substantially reduced following retrieval of NH_3_. (The second strong residual feature near 949 cm^−1^ in panels b and c appears to be due to the ν_7_ Q-branch of ethylene (C_2_H_4_), as is discussed in Section 3.4 below.)

**Figure 2.**
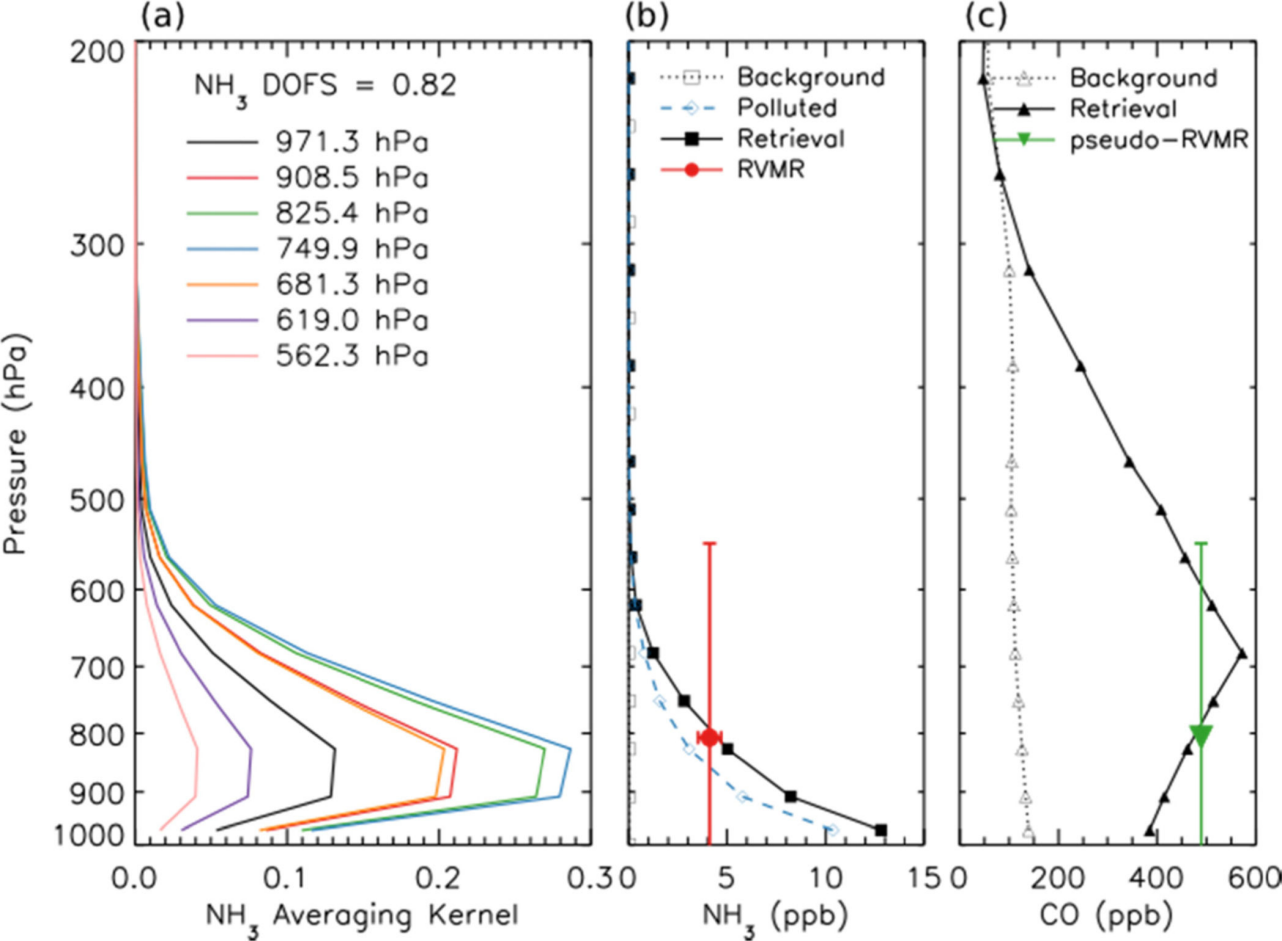
(**a**) NH_3_ averaging kernel for the same TES scan as shown in Figure 1. (**b**) Background unpolluted NH_3_ profile (open black squares), polluted *a priori* NH_3_ profile (open blue diamonds), retrieved NH_3_ profile (filled black squares) and NH_3_ Representative Volume Mixing Ratio (RVMR) (red circle) for the same scan. The horizontal error bars show the uncertainty in the RVMR due to noise in the TES spectrum, while the vertical bars encompass the region of TES sensitivity to NH_3_. (**c**) Background (and *a priori*) CO profile (open black triangles), retrieved CO profile (filled black triangles) and CO pseudo-RVMR (green inverted triangle) for the same scan.

Figure 2a shows the averaging kernel for the same NH_3_ retrieval. The retrievals are performed using 14 vertical levels. However, the number of DOFS is generally not greater than one, meaning that it is not possible to obtain information about the shape of the profile from these retrievals. In order to minimize the influence of the *a priori* constraints on the end result, it is desirable to report one retrieved quantity per DOFS. However, performing a “profile” retrieval offers a major advantage in that us allows the calculation of diagnostics, such as the averaging kernels, that enable characterization of the spatial and temporal variability of the vertical sensitivity of the measurements. Therefore, for this work, the retrievals are performed on 14 levels in order to take advantage of the sensitivity characterization that this enables, then post-processed to calculate a single quantity that better represents the information that is really available in the measurement and that is relatively insensitive to the *a priori* constraints. Shephard *et al.* developed a Representative Volume Mixing Ratio (RVMR) metric for NH_3_ [20] based on similar techniques used previously for CH_4_ [51] and CH_3_OH [19]. This RVMR represents a TES sensitivity weighted average value where the influence of the *a priori* profile is reduced as much as possible. (Note that for NH_3_, one of three *a priori* profiles is selected based on the signal-to-noise ratio in the TES NH_3_ band [20]. For the scan shown in Figures 1 and 2, the polluted *a priori*, shown as a dashed blue line in Figure 2b, was selected.) Figure 2b shows a retrieved profile of NH_3_ for a fresh Canadian smoke plume as a solid black line, while the red circle is the RVMR calculated from the profile. The horizontal error bars show the uncertainty in the RVMR due to noise in the TES spectrum, while the vertical bars encompass the region of TES sensitivity to NH_3_, defined as the full width at half maximum (FWHM) of the averaging kernel at the pressure level of the RVMR [20]. With the *a priori* assumption about profile shape that was chosen here the NH_3_ RVMR is roughly 20% to 60% of the retrieved surface value for NH_3_. The estimated minimum detection level is an RVMR of approximately 0.3 ppb, corresponding to a profile with a surface mixing ratio of about 1–2 ppb [20]. However, it must be noted that this is merely a general estimate of the minimum detection level, which depends strongly on the location of peak NH_3_ mixing ratio and the thermal structure of the atmosphere for a given scan [20], and thus retrieved NH_3_ RVMRs of less than 0.3 ppb are considered valid and are included in our analysis.

The excess mixing ratio of a trace gas like NH_3_ (EMR, ΔNH_3_) is defined as the mixing ratio of the gas in the smoke plume minus its mixing ratio in the background. This excess mixing ratio can be normalized using the excess mixing ratio of CO to give the normalized excess mixing ratio (NEMR, ΔNH_3_/ΔCO). The emission ratio (ER) is a special case of the NEMR where the measurements are made in fresh smoke near the fire source [3]. The NEMR of a trace gas can be highly variable for reactive gases downwind of fires due to the different rates of deposition and secondary photochemical production and loss for the trace gas and CO. In this paper, we will refer to our derived NEMRs for fresh Canadian smoke observed by TES as emission ratios; however, these emission ratios are inherently convolutions of the initial NEMR and any secondary production and loss processes that have taken place within the smoke plume during plume lofting and transport from the fire source [28]. Furthermore, the lower sensitivity of the TES retrievals near the surface (see Figure 2a) means that TES will preferentially sample smoke from the flaming stages of combustion, as these emissions are more likely to be lofted well above the surface. This lower sensitivity near the surface is due to the physics of nadir thermal infrared sounding: when the surface and the layers of the atmosphere near the surface have similar temperatures (low thermal contrast), we cannot distinguish between radiation emitted by the surface and radiation emitted by the lowest layers of the atmosphere. Similar caution must be used in interpreting emission ratios measured from other platforms: for example, NEMRs of NH_3_ measured at the ground are generally much higher than those measured by aircraft, as the aircraft does not sample the emissions from residual smoldering combustion very close to the ground (see Section 3.1 below).

In order to use the TES retrievals of NH_3_ and CO to calculate the emission ratio of NH_3_, we first calculated the RVMR for NH_3_ following the procedure of Shephard *et al.* [20]. Since the DOFS for the CO retrieval are generally higher than for NH_3_, in order to obtain a comparable metric we transform the TES CO retrieval using the same grid and weightings as were used to generate the NH_3_ RVMR to obtain a pseudo-RVMR for CO. Figure 2c shows the retrieved CO profile for a fresh Canadian smoke plume as a solid black line while the pseudo-RVMR for CO is shown in green. The emission ratio of NH_3_ was then calculated as the slope of a least squares linear regression of the NH_3_ RVMR and the CO pseudo-RVMR.

### 2.3. HCOOH Retrieval

**Figure 3.**
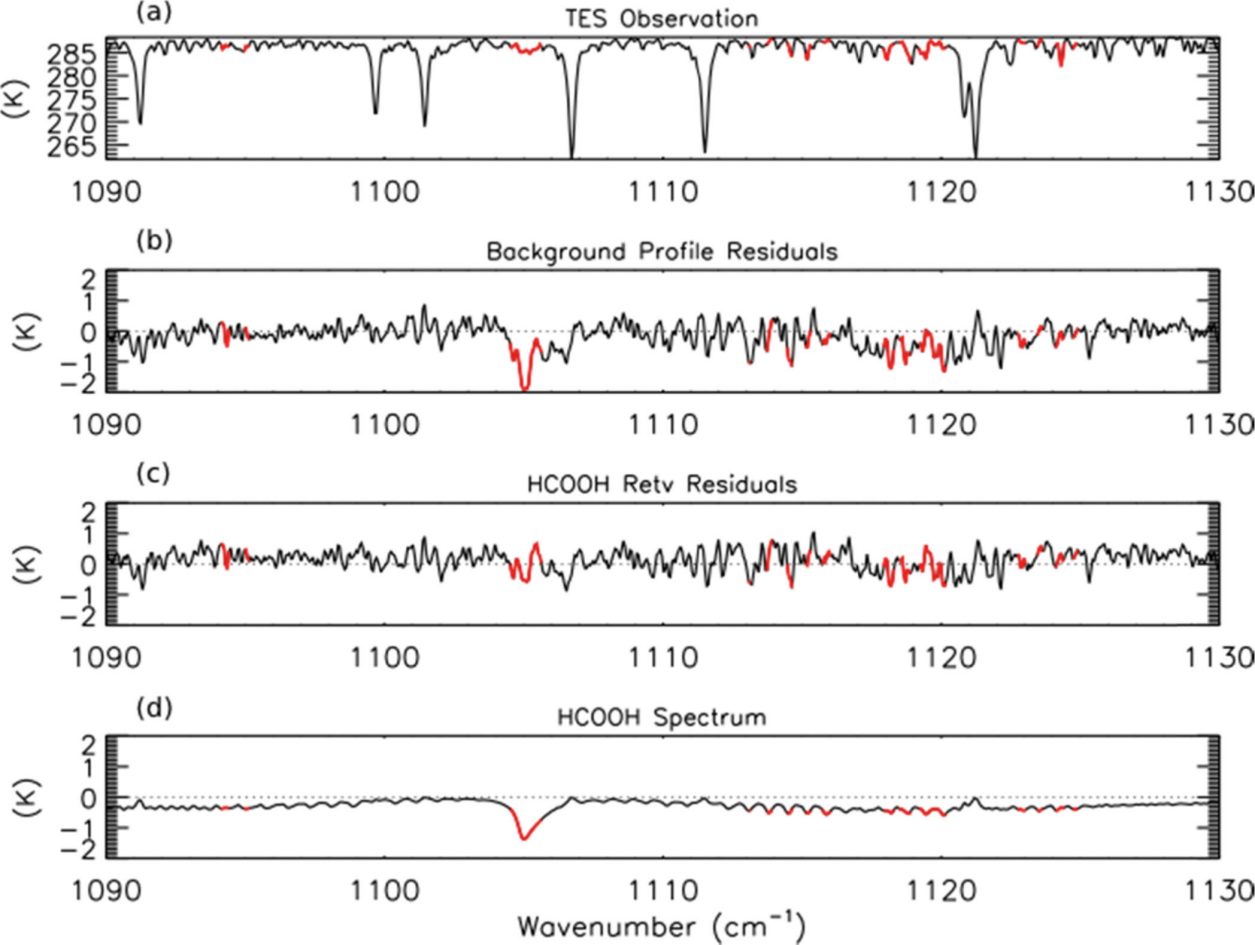
(**a**) TES observed brightness temperature spectrum, (**b**) background formic acid (HCOOH) profile residuals, calculated as observed minus modeled spectrum, (**c**) residuals after retrieval of HCOOH, and (**d**) the modeled spectrum of HCOOH for the same TES scan as shown in Figure 1. The microwindows used in the HCOOH retrieval are shown in red.

We have developed a prototype retrieval for formic acid (HCOOH) from TES Level 1B spectra. The retrieval approach is similar to that used for NH_3_. The spectroscopic parameters for HCOOH were taken from the HITRAN 2008 database, which substantially improved the estimates of the strengths of HCOOH lines by removing interference from the formic acid dimer [52]. All other spectroscopic parameters were taken from v1.4 of the TES spectroscopic line parameters [53]. The *a priori* constraint and covariance matrix were compiled from GEOS-Chem model simulations of the 2004 global background mixing ratios of HCOOH; however, these background profiles may be biased low, as GEOS-Chem tends to underestimate HCOOH in the northern mid-latitudes [28]. For HCOOH, the initial guess profile is the same as the *a priori* constraint vector. LBLRTM was run using TES Level 2 (V004) retrievals of temperature, average cloud optical depth, emissivity, reflectivity, H_2_O, CO, O_3_, and CH_4_. Profiles of CO_2_ and N_2_O were taken from the TES Level 2 supplemental data files. The top panel of Figure 3 shows the observed TES brightness temperature spectrum between 1090–1130 cm^−1^ for a scan of fresh smoke from a Canadian fire with the HCOOH retrieval microwindows shown in red. Note that only some sections of the HCOOH band have been retained in order to remove interfering lines from other species, principally water vapor. The second panel shows the residuals (observed spectrum minus modeled spectrum) for the background HCOOH profile. We see a strong (−2 K) residual in the HCOOH Q-branch (~1105 cm^−1^), which is removed after retrieval of HCOOH as seen in the third panel of Figure 3.

**Figure 4.**
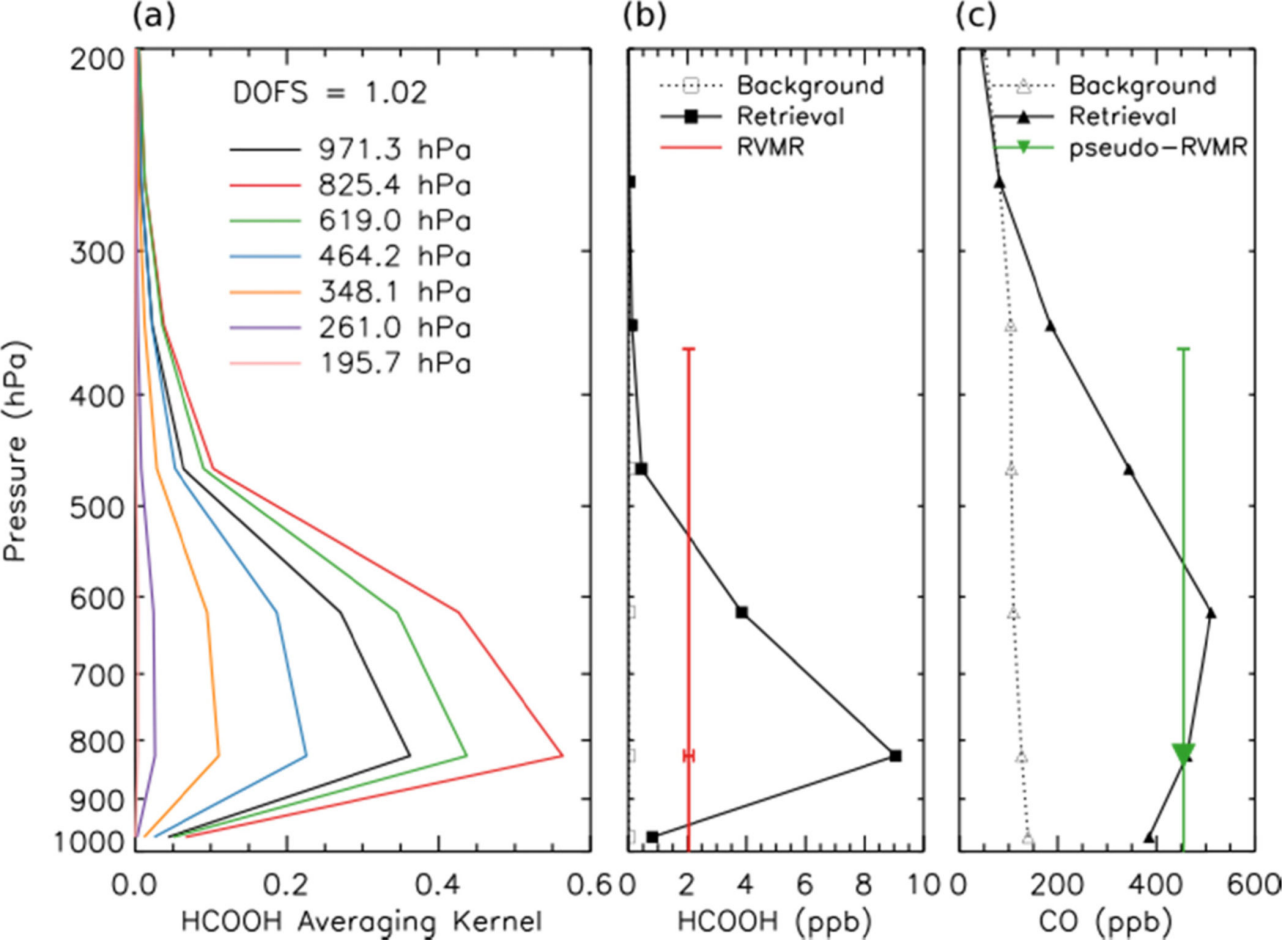
(**a**) HCOOH averaging kernel for the same TES scan as shown in Figure 1. (**b**) Background (and *a priori*) HCOOH profile (open black squares), retrieved HCOOH profile (filled black squares), and HCOOH RVMR (intersection of red lines) for the same scan. The red dot was omitted so that the horizontal error bars would be visible. (**c**) Background (and *a priori*) CO profile (open black triangles), retrieved CO profile (filled black triangles) and CO pseudo-RVMR (green inverted triangle) for the same scan.

An RVMR for HCOOH and a corresponding CO pseudo-RVMR were calculated following the procedure of Shephard *et al.* for NH_3_ [20], as illustrated in Figure 4. The RVMR for HCOOH is lower than the retrieved value at the level of maximum sensitivity to HCOOH, possibly because the TES sensitivity to HCOOH covers a wide pressure range. The emission ratio of HCOOH was calculated as the slope of the least squares linear regression of the HCOOH RVMR and the CO pseudo-RVMR.

### 2.4. Detection of PAN and C_2_H_4_

In order to use TES to detect PAN in boreal smoke plumes, we calculated the differences (residuals) between the TES Level 1B spectra (V003) and a forward run of LBLRTM using v1.4 of the TES spectroscopic line parameters for the region of strong absorption by PAN (1140–1180 cm^−1^). As for the retrievals of HCOOH, the model was run using TES Level 2 (V004) retrievals of temperature, emissivity, reflectivity, average cloud optical depth, H_2_O, CO, O_3_, and CH_4_ and profiles of CO_2_ and N_2_O were taken from the TES Level 2 supplemental data files. Preliminary model runs using the spectrally resolved effective cloud optical depth retrieved by TES led to unphysical slopes in the residuals versus wavenumber between 1100 and 1200 cm^−1^. We removed these slopes by setting cloud optical depth in this spectral region to the average effective cloud optical depth included in the TES Level 2 products; however, the spectral signature of PAN was detectable regardless of which TES cloud product was used. The absorption cross section of PAN was taken from HITRAN 2008 [52].

A similar procedure was used to detect C_2_H_4_ in TES spectra, with the addition that the surface emissivity, surface temperature, and NH_3_ profile were taken from the final results of the NH_3_ retrieval described in Section 2.2 above. The line parameters for C_2_H_4_ were taken from v1.4 of the TES spectroscopic line parameters.

## 3. Results and Discussion

### 3.1. Emission Ratio of NH_3_

**Figure 5.**
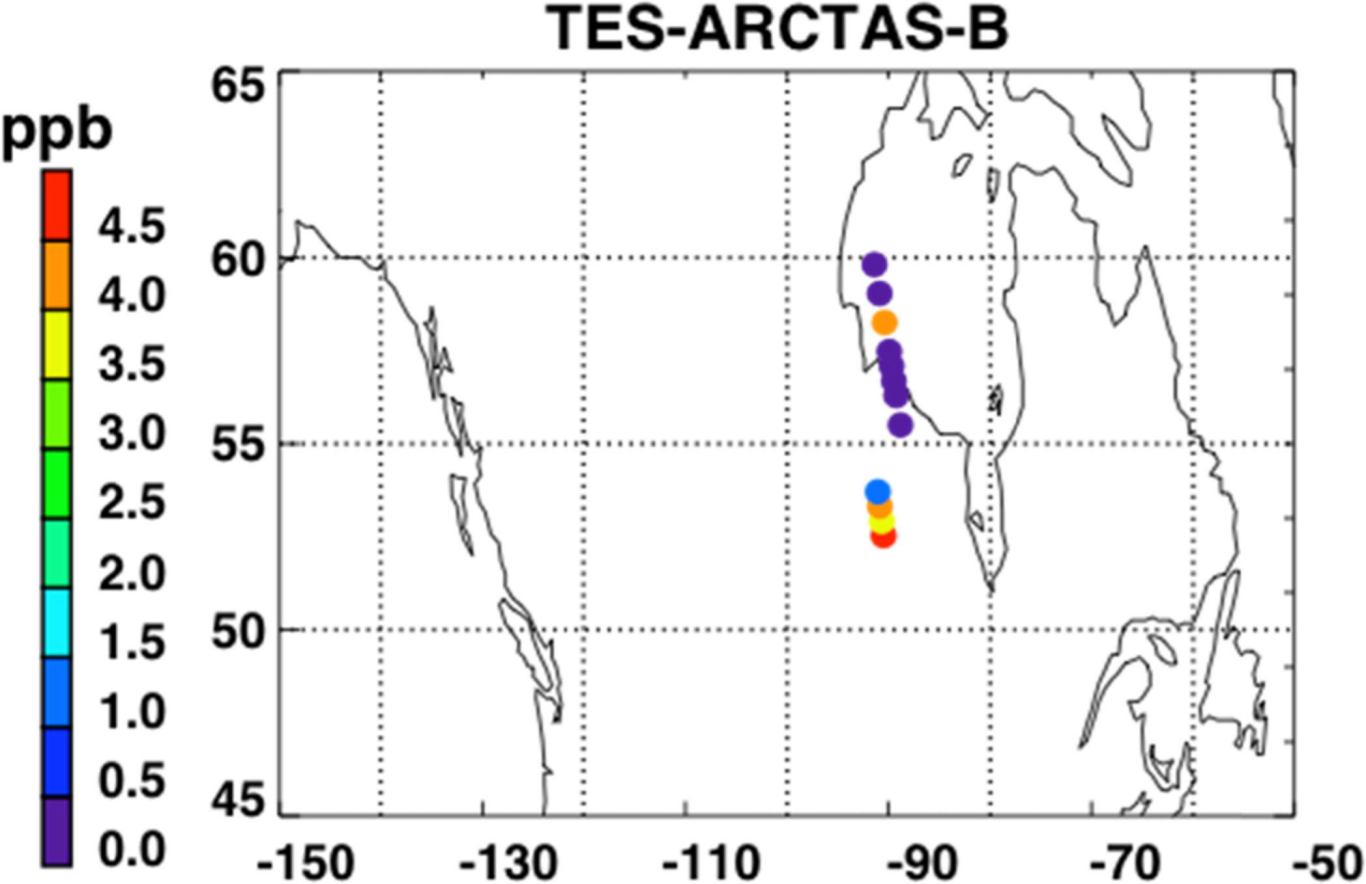
RVMR of NH_3_ for the TES special observations for Arctic Research of the Composition of the Troposphere from Aircraft and Satellites (ARCTAS-B) between 15 June 2008 and 15 July 2008. Only retrievals within biomass burning plumes over Canada are shown. Note that the red point is 7.3 ppb.

Figure 5 shows a map of the retrieved NH_3_ RVMR values within smoke plumes from Canadian biomass burning in the summer of 2008. We have filtered the retrievals for quality following Shephard *et al.* [20]. First, NH_3_ retrievals are rejected if the DOFS are less than 0.1 or if the average cloud optical depth is greater than 1. Second, retrievals with DOFS less than 0.5 are rejected if the thermal contrast between the surface and the atmospheric layer nearest the surface is between −7 K and 10 K.

**Figure 6.**
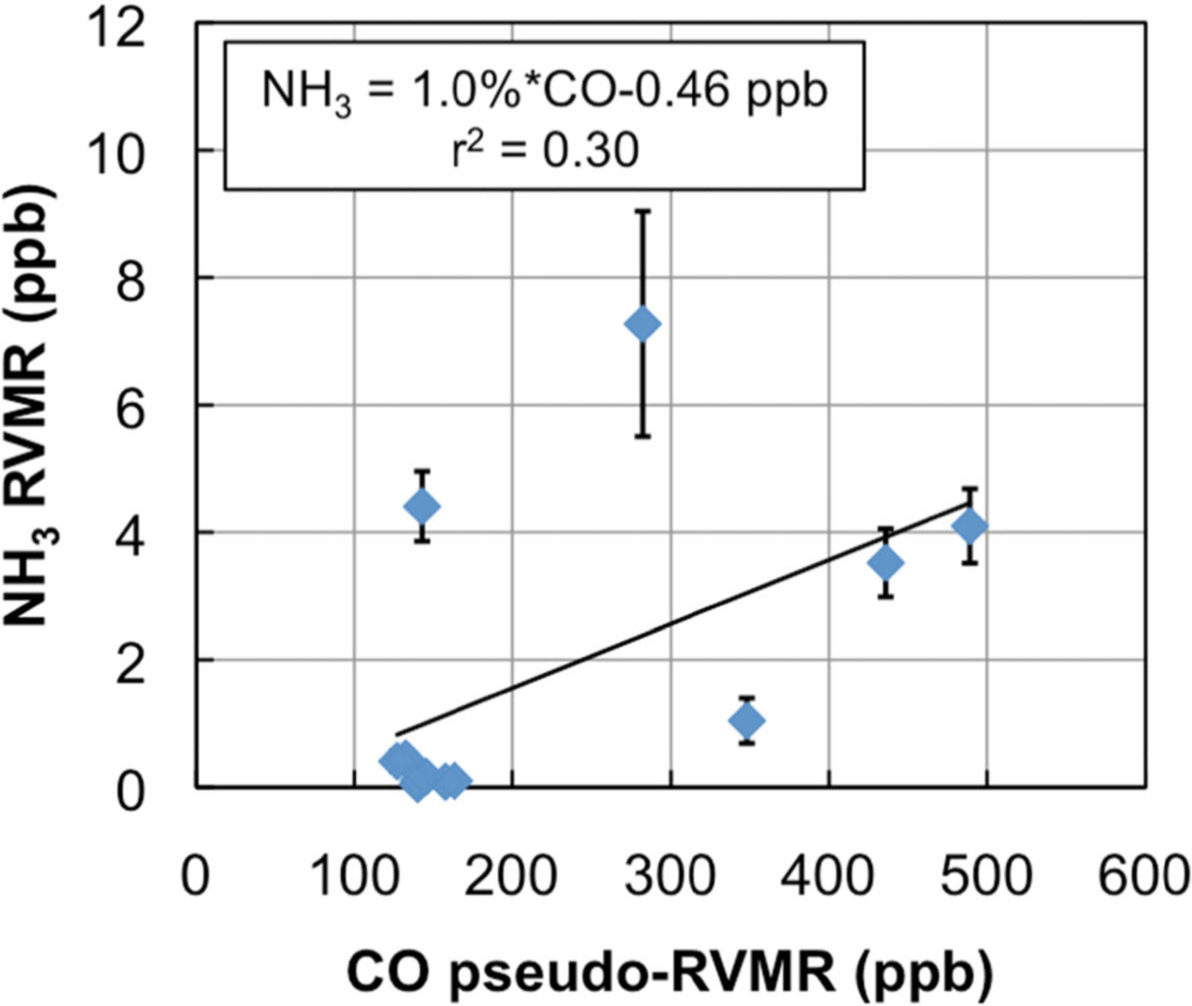
RVMR of NH_3_ versus the pseudo-RVMR of CO for all of the retrievals shown in Figure 5.

Figure 6 shows the NH_3_ RVMR versus the CO pseudo-RVMR for all of the retrievals shown on the map in Figure 5. The most elevated amounts of CO and NH_3_ come from a fresh smoke plume from a single Canadian fire observed by TES between 52.5–54.1°N and 90.5–91.8°W at 19:03 UTC on 1 July 2008 (TES Run #7656). Due to the high retrieved values for CO (>500 ppb), this plume likely contains concentrated fresh smoke. Also included are retrievals of NH_3_ within a more dilute smoke plume observed between 55.5–59.8°N and 88.8–91.4°W at 18:51 UTC on 17 June 2008 (TES Run #7472).

We calculate the NH_3_ emission ratio (ΔNH_3_/ΔCO) for these fires as 1.0% ± 0.5%. However, there is a large amount of scatter in this plot, which leads to a low correlation between CO and NH_3_ (*r*^2^ = 0.30). The scatter is likely due to the combination of: (1) variations in plume heights, which change the relative sensitivity of TES to CO and NH_3_ in the smoke plume; (2) variations in the age of the smoke plumes, as older plumes could have lost more NH_3_ to chemical reaction or deposition, and (3) variations in the initial emissions of ammonia from the fires, which are in turn related to differences in the relative fraction of flaming versus smoldering combustion as well as variations in fuel nitrogen content.

Table 1 compares our derived emission ratio for NH_3_ to previous satellite, aircraft, and ground measurements of ammonia emission ratios for boreal biomass burning. Our value is close to the value of 1.3% derived from IASI retrievals in Siberian smoke over East Mongolia [9]. The results from the aircraft and ground studies are substantially different with ground studies showing values of ΔNH_3_/ΔCO that are a factor of 5 higher than the aircraft or satellite studies. The higher ground values are likely due to emissions of NH_3_ from residual smoldering combustion very close to the ground [3] where aircraft cannot sample and satellites have little sensitivity to NH_3_ (see Figure 2). Thus, both aircraft and satellites are likely to underestimate the true emissions of NH_3_ from biomass burning fires unless the emission factors from aircraft and satellite studies are carefully combined with ground observations, such as in the review of Akagi *et al.* [3]. In addition, given the discrepancy between aircraft and ground observations, nadir-viewing satellite observations of NH_33_ are most appropriately compared with aircraft observations, as satellites will not be very sensitive to the ground-level residual smoldering emissions detected in ground studies. Our NH_3_ emission factor of 1.0% ± 0.5% is similar to the values of 1.3% ± 0.7% and 1.7% ± 0.9% reported for Alaskan forest fires in the aircraft studies of Nance *et al.* [54] and Goode *et al.* [55], respectively, but is substantially higher than the value of 0.16% ± 0.14% reported by Radke *et al.* for boreal forests [56]. Furthermore, our value lies within the uncertainty range of the average value of 1.0% ± 0.7% (boreal forests, aircraft studies only) reported in the review of Akagi *et al.* [3], as well as the value of 2.6% ± 2.2% (all extratropical forests, all study types) in the review of Andreae and Merlet [57].

### 3.2. Emission Ratio of HCOOH

**Figure 7.**
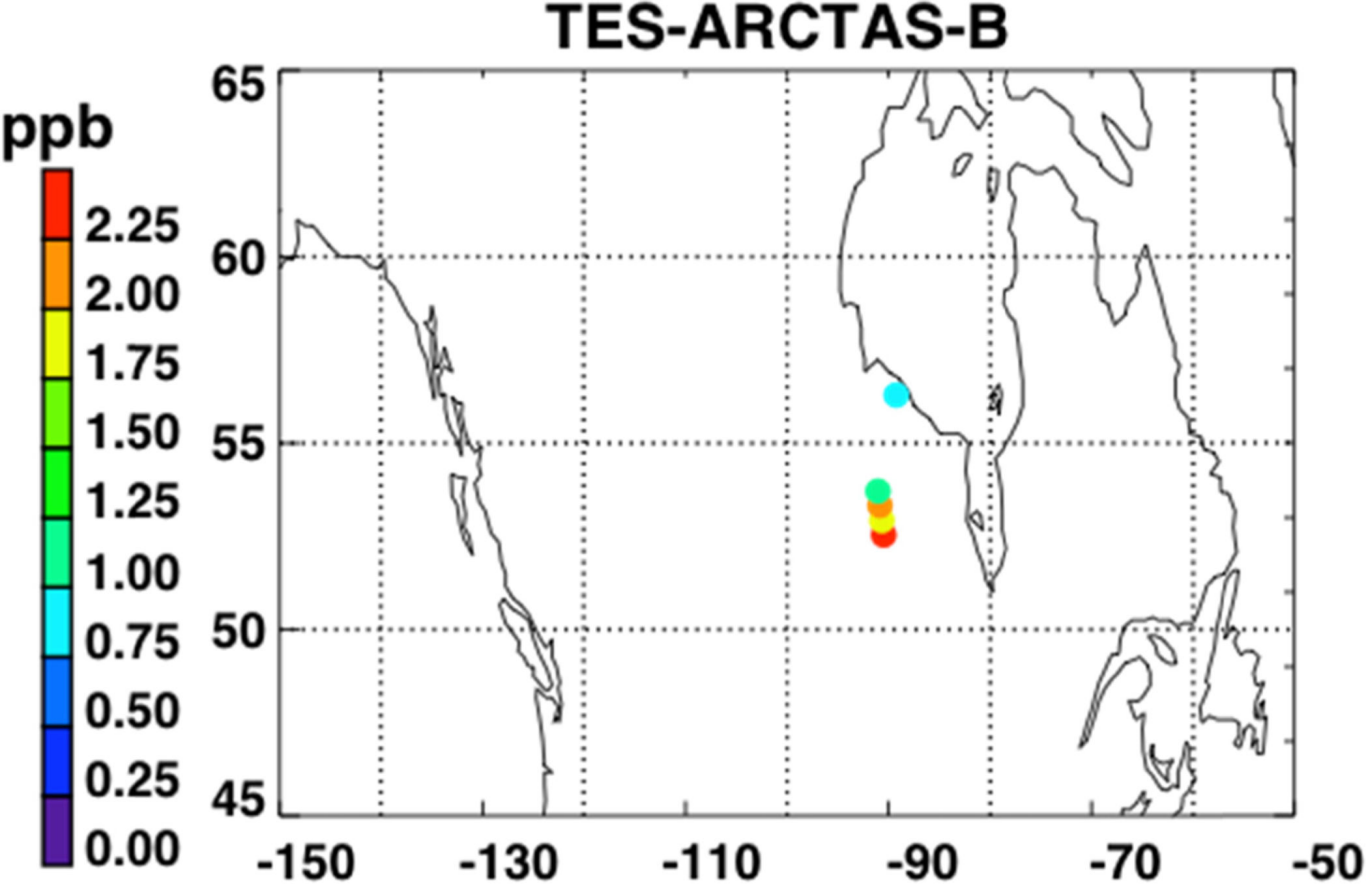
RVMR of HCOOH for the TES special observations for ARCTAS-B between 15 June 2008 and 15 July 2008. Only retrievals within biomass burning plumes over Canada are shown.

**Figure 8.**
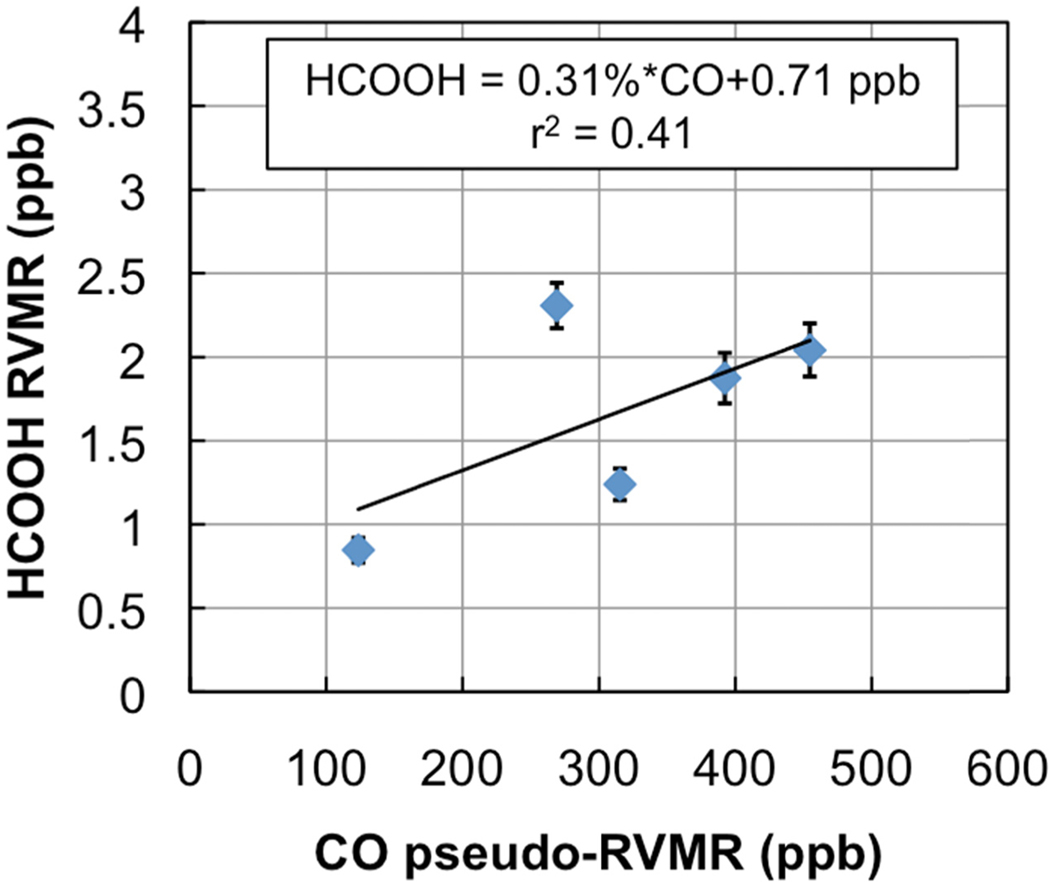
RVMR of HCOOH versus the pseudo-RVMR of CO for the retrievals shown in Figure 7.

Figure 7 shows a map of the retrieved HCOOH RVMR values within smoke plumes from boreal biomass burning over Canada. We have removed retrievals with less than 0.5 DOFS for HCOOH, which also eliminated retrievals with an HCOOH RVMR of less than 0.5 ppbv. The smoke plumes included in the analysis are the same as described above for NH_3_. Figure 8 shows the HCOOH RVMR versus the CO pseudo-RVMR for all of the retrievals shown on the map in Figure 7. We calculate the emission ratio ΔHCOOH/ΔCO for these fires as 0.31% ± 0.21%. As we saw for NH_3_, there is a large amount of scatter in this plot that leads to a low correlation between CO and HCOOH (*r*^2^ = 0.41). As discussed above, this scatter is likely due to the combination of variations in (1) plume heights, (2) plume ages, and (3) initial smoke emissions of HCOOH. The large pressure range of TES sensitivity to HCOOH may also contribute to the low correlation of HCOOH with CO. It is worth noting that the ACE-FTS observations of HCOOH within boreal biomass burning plumes showed a much higher *r*^2^ value (0.86), likely due to the fact that the higher vertical resolution of limb retrievals means that ACE is better able to separate the different vertical layers of the plume which are averaged together by nadir-viewing sounders like TES and IASI [31].

Table 2 compares our derived emission ratio for HCOOH to previous satellite, aircraft, and ground measurements of formic acid emission ratios for boreal biomass burning. Our HCOOH emission ratio is slightly lower than the ratio of 0.38% ± 0.06% derived from ACE-FTS observations [31], possibly due to the fact that a limb-sounder like ACE is more sensitive to plumes that reach high altitudes; this type of plume is more common over more flaming fires since fire power output peaks during flaming combustion [4]. Table 2 shows that our value is similar to the emission ratios for HCOOH derived from aircraft and ground studies, with the exception of the value of 3.7% ± 2.0% reported for Canadian forests by Lefer *et al.* [60], which is about a factor of 10 higher than the average value for boreal biomass burning in the review of Akagi *et al.* [3]. However, Lefer *et al.* note that the elevated enhancement ratios of HCOOH they observed (0.82% to 6.2%) are likely due to HCOOH production within the plumes in the first couple hours after emission, and do not necessarily reflect the initial emissions. When the Lefer *et al.* study is removed, the Andreae and Merlet average becomes 0.85% ± 0.53%, with the relatively higher value of HCOOH likely due to the inclusion of midlatitude fires as well as boreal fires in the average [57].

### 3.3. Detection of PAN

**Figure 9.**
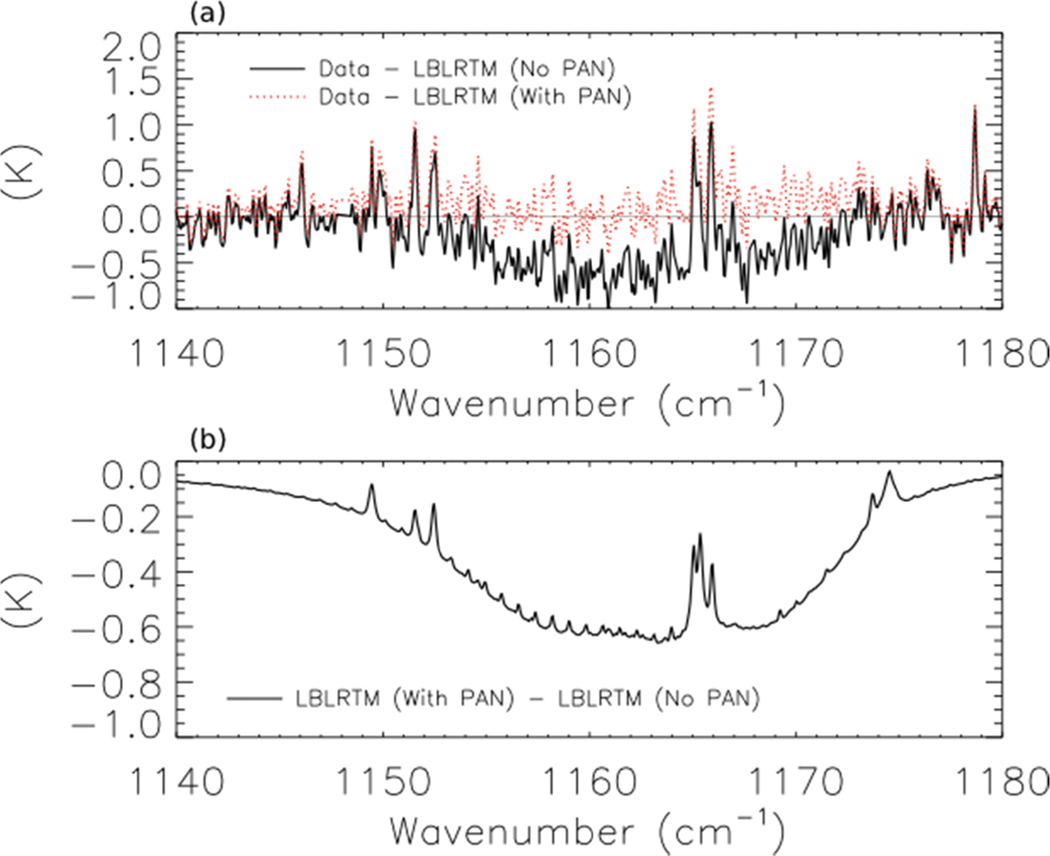
(**a**) Brightness temperature residuals (data minus model) for the same TES scan as in Figure 1. The solid black line does not have PAN in the forward model, while the dotted red line does include PAN. (**b**) Difference between the model runs with and without PAN, which shows the modeled spectrum of PAN.

The solid black line of Figure 9a shows the brightness temperature residuals (data minus model) for the scan of fresh smoke from a Canadian fire at 53.32°N and 90.84°W.

The two lobes of PAN absorption are clearly visible on either side of the water line at ~1165 cm^−1^. The dotted red line shows the residuals when a hypothetical PAN profile with a peak concentration of 1.9 ppb at 560 hPa is added to the forward model, and Figure 9b shows the modeled PAN spectrum as a solid black line. Adding the PAN profile substantially reduces the mean residuals, but this peak concentration of PAN in the assumed profile is about a factor of 2 higher than the concentration of PAN observed 30–60 min downwind of the Lake McKay fire in Saskatchewan, Canada during ARCTAS-B [12]. We have also detected PAN in a scan of aged smoke from Siberian biomass burning observed near Kamchatka (not shown). These two scans shared several common features, including relatively high mixing ratios of CO (mixing ratio > 250 ppbv at 510 hPa) and low average cloud optical depth (<0.1). This low cloud optical depth may be a necessary but insufficient criterion for detecting PAN in biomass burning smoke plumes with TES.

### 3.4. Detection of C_2_H_4_

As mentioned in Section 2.2 above, there is a second strong residual feature in Figure 1c near 949 cm^−1^ that appears to be due to absorption by C_2_H_4_.

**Figure 10.**
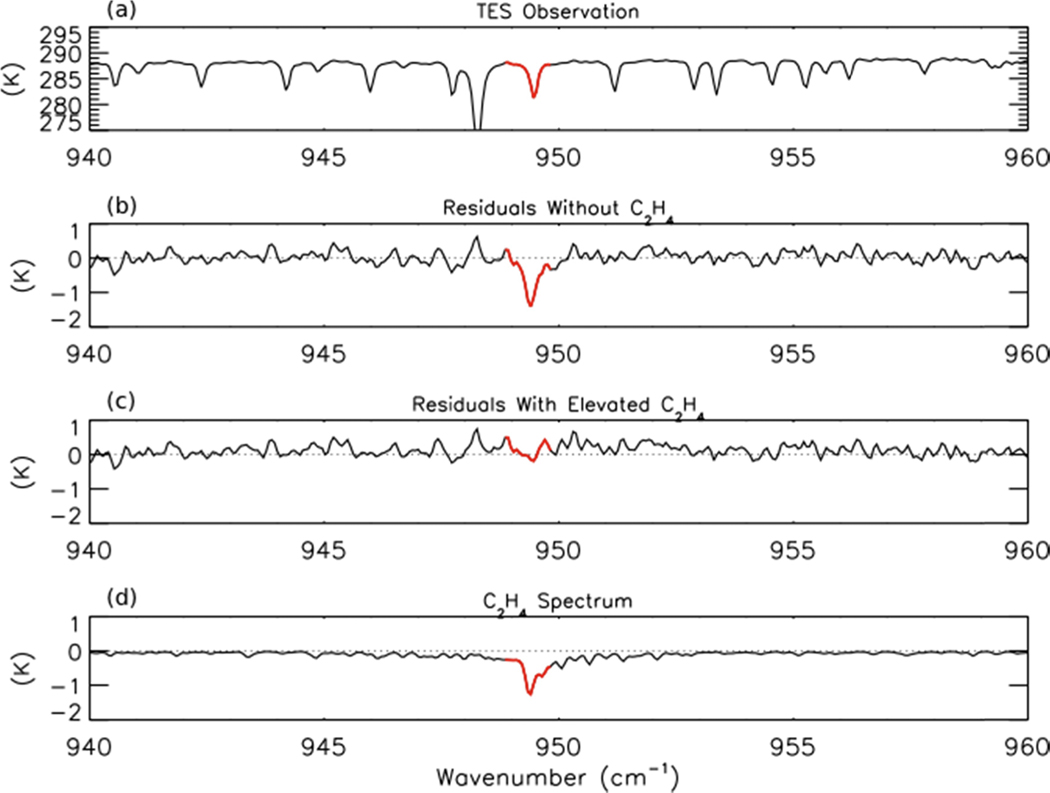
(**a**) TES observed brightness temperature spectrum, (**b**) residuals without a C_2_H_4_ profile in the model, calculated as observed minus modeled spectrum, (**c**) residuals after addition of a hypothetical profile of C_2_H_4_, and (**d**) the modeled spectrum of C_2_H_4_ for the same TES scan as shown in Figure 1. The area of strong absorption by C_2_H_4_, corresponding to the ν_7_ Q-branch, is shown in red.

Figure 10b shows this residual feature, which partially overlaps with the strong CO_2_ line [39] that is visible in the TES spectrum shown in Figure 10a. Figure 10c shows that the addition of a hypothetical C_2_H_4_ profile to the model (with a peak concentration of 1.9 ppb at the surface) removes this feature. This peak C_2_H_4_ mixing ratio is approximately the 85th percentile of C_2_H_4_ observations observed downwind of the Lake McKay fire in Saskatchewan, Canada during ARCTAS-B [12]. While the scan in Figure 10 did have a low cloud optical depth, this criterion is likely to be less important for detecting C_2_H_4_ within fresh biomass burning plumes using TES, as the residual feature of C_2_H_4_ is both stronger and sharper than the PAN feature. However, the short lifetime of C_2_H_4_ may make it difficult to detect in smoke that is several hours old.

## 4. Conclusions

We have retrieved mixing ratios of ammonia (NH_3_) and formic acid (HCOOH) within biomass burning smoke plumes over Canada from TES radiance measurements. We have combined these retrievals with the TES retrievals of CO to calculate molar ratios of NH_3_ and HCOOH to CO within biomass burning plumes by calculating representative volume mixing ratios for NH_3_ and HCOOH and then mapping the CO retrieval to the same vertical grid. Our estimated emission ratios for NH_3_ (1.0% ± 0.5%) and HCOOH (0.31% ± 0.21%) for forest fires in Canada are within the range of values reported in the literature for airborne and satellite studies of boreal biomass burning emissions. This work thus provides a method for the use of TES spectra to study the emissions of NH_3_ and HCOOH from biomass burning. This method, if applied to the entire TES data set, would help to estimate the spatial and temporal variability of these emissions. This information could then be used, in concert with the other satellite observations discussed in Section 1 of our manuscript, to provide improved estimates of the emissions of these trace gases for use in models of atmospheric chemistry, air quality, and climate.

We have shown that TES can observe peroxy acetyl nitrate (PAN) and ethylene (C_2_H_4_) within boreal biomass burning plumes. A low cloud optical depth (<0.1) appears to be required for successful detection of PAN by TES within biomass burning smoke plumes. Continuing this line of research could lead to maps of tropospheric PAN concentrations near source regions, which would help to constrain the fate of NO_x_ emission within atmospheric chemistry models. These could be combined with satellite estimates of the emissions of ethylene from biomass burning to help reduce the uncertainty in the impact of biomass burning on tropospheric ozone.

## Figures and Tables

**Table 1.** Ammonia emission ratios (ΔNH_3_/ΔCO, mol/mol) for boreal biomass burning.

ΔNH_3_/ΔCO	Ecosystem/Fuel	Study Type	Source

1.0% ± 0.5%	Canadian Forest	Satellite (TES)	This Study
1.3%	Siberian Forest	Satellite (IASI)	[9]
0.16% ± 0.14%	Boreal Forests	Aircraft	[56]
1.3% ± 0.7%	Alaskan Forest	Aircraft	[54]
1.7% ± 0.9%	Alaskan Forest	Aircraft	[55]
6.9% ± 11.1%	Boreal Peat	Ground	[58]
5.9% ± 3.6%	Boreal organic soil	Ground	[58]
6.9% ± 3.2%	Boreal organic soil	Ground	[59]
1.7% ± 2.2%	Boreal dead, woody material	Ground	[59]
3.5%	Alaskan Duff	Laboratory	[4]
1.0% ± 0.7%	Boreal Forests	Review: Aircraft Only	[3]
5.1% ± 5.6%	Boreal Forests	Review: Ground Only	[3]
3.5% ± 3.2%	Boreal Forests	Review: Aircraft and Ground	[3]
2.6% ± 2.2%	Extratropical Forests	Review: Aircraft, Satellite, and Ground	[57]

**Table 2.** Formic acid emission ratios (ΔHCOOH/ΔCO, mol/mol) for boreal biomass burning.

ΔHCOOH/ΔCO	Ecosystem/Fuel	Study Type	Source

0.31% ± 0.21%	Canadian Forest	Satellite (TES)	This Study
0.38% ± 0.06%	Canadian Forest	Satellite (ACE-FTS)	[31]
0.31% ± 0.09%	Alaskan Forest	Aircraft	[55]
0.26% ± 0.44%	Boreal Peat	Ground	[58]
0.18% ± 0.12%	Boreal organic soil	Ground	[58]
0.33% ± 0.29%	Boreal organic soil	Ground	[59]
0.17% ± 0.18%	Boreal dead, woody material	Ground	[59]
0.35%	Alaskan Duff	Laboratory	[4]
0.28% ± 0.24%	Boreal Forests	Review, Aircraft and Ground	[3]
3.7% ± 2.0%	Canadian Forest	Aircraft	[60]
1.4% ± 1.4%	Extratropical Forests	Review, Aircraft, Satellite and Ground	[57]
